# Three-Day Monitoring of Adhesive Single-Lead Electrocardiogram Patch for Premature Ventricular Complex: Prospective Study for Diagnosis Validation and Evaluation of Burden Fluctuation

**DOI:** 10.2196/46098

**Published:** 2024-03-21

**Authors:** Hyo-Jeong Ahn, Eue-Keun Choi, So-Ryoung Lee, Soonil Kwon, Hee-Seok Song, Young-Shin Lee, Seil Oh

**Affiliations:** 1 Department of Internal Medicine Seoul National University Hospital Seoul Republic of Korea; 2 Department of Internal Medicine Seoul National University College of Medicine Seoul Republic of Korea; 3 Seers Technology Co, Ltd Seongnam-si Gyeonggi-do Republic of Korea

**Keywords:** premature ventricular complex, single-lead electrocardiogram, wearable device, extended monitor, electrocardiogram, EKG, ECG, wearable, heart, cardiology, cardiovascular

## Abstract

**Background:**

Wearable electrocardiogram (ECG) monitoring devices are used worldwide. However, data on the diagnostic yield of an adhesive single-lead ECG patch (SEP) to detect premature ventricular complex (PVC) and the optimal duration of wearing an SEP for PVC burden assessment are limited.

**Objective:**

We aimed to validate the diagnostic yield of an SEP (mobiCARE MC-100, Seers Technology) for PVC detection and evaluate the PVC burden variation recorded by the SEP over a 3-day monitoring period.

**Methods:**

This is a prospective study of patients with documented PVC on a 12-lead ECG. Patients underwent simultaneous ECG monitoring with the 24-hour Holter monitor and SEP on the first day. On the subsequent second and third days, ECG monitoring was continued using only SEP, and a 3-day extended monitoring was completed. The diagnostic yield of SEP for PVC detection was evaluated by comparison with the results obtained on the first day of Holter monitoring. The PVC burden monitored by SEP for 3 days was used to assess the daily and 6-hour PVC burden variations. The number of patients additionally identified to reach PVC thresholds of 10%, 15%, and 20% during the 3-day extended monitoring by SEP and the clinical factors associated with the higher PVC burden variations were explored.

**Results:**

The recruited data of 134 monitored patients (mean age, 54.6 years; males, 45/134, 33.6%) were analyzed. The median daily PVC burden of these patients was 2.4% (IQR 0.2%-10.9%), as measured by the Holter monitor, and 3.3% (IQR 0.3%-11.7%), as measured in the 3-day monitoring by SEP. The daily PVC burden detected on the first day of SEP was in agreement with that of the Holter monitor: the mean difference was –0.07%, with 95% limits of agreement of –1.44% to 1.30%. A higher PVC burden on the first day was correlated with a higher daily (*R*^2^=0.34) and 6-hour burden variation (*R*^2^=0.48). Three-day monitoring by SEP identified 29% (12/42), 18% (10/56), and 7% (4/60) more patients reaching 10%, 15%, and 20% of daily PVC burden, respectively. Younger age was additionally associated with the identification of clinically significant PVC burden during the extended monitoring period (*P*=.02).

**Conclusions:**

We found that the mobiCARE MC-100 SEP accurately detects PVC with comparable diagnostic yield to the 24-hour Holter monitor. Performing 3-day PVC monitoring with SEP, especially among younger patients, may offer a pragmatic alternative for identifying more individuals exceeding the clinically significant PVC burden threshold.

## Introduction

Premature ventricular complexes (PVCs) are commonly observed in individuals who have undergone long-term ambulatory monitoring [1]. Frequent PVCs can result in reversible cardiomyopathy, although patients with PVCs without underlying structural heart disease are usually expected to have a benign clinical prognosis [2-4].

Several risk factors are known to contribute to the overall risk of developing PVC-induced cardiomyopathy (PIC), and the burden of PVC is one of the most important predictors of clinical deterioration [1,5,6]. Although there is no absolute cutoff of PVC burden to identify patients at risk of developing PIC, 16%-26% of PVC burden is reported as a significant threshold to develop PIC [5,7,8]. Of note, at least 10% of the PVC burden is considered sufficient for developing PIC to warrant a regular assessment of structural and functional cardiac change [9].

For the determination of an appropriate treatment strategy for PVC, one of the essential pieces of information that should be accurately assessed is the PVC burden, together with the accompanying symptoms and the presence or absence of structural heart disease. However, defining the exact PVC burden remains a challenge because of its substantial hourly or daily variation, and recent evidence recommends that patients undergo ambulatory ECG monitoring throughout the day to observe its maximum burden [1]. The 24-hour Holter monitoring has been regarded as the gold standard for evaluating PVC burden, but the Holter monitor is uncomfortable to wear, and the 24-hour duration is insufficient to appreciate the substantial burden variation demonstrated among individuals [10,11].

With the advent of widespread use of wearable electrocardiogram (ECG) patch monitoring, studies have suggested that extended monitoring is required to estimate more accurate daily PVC burden [10,12]. Recently, an adhesive single-lead ECG patch (SEP) has been found to enable more convenient extended monitoring to capture fluctuations in the PVC burden, but the diagnostic yield of SEP has not been thoroughly validated. Moreover, the optimal duration of ambulatory ECG monitoring for PVC evaluation has not been determined, and the clinical significance of 3-day SEP monitoring (a shorter period than studied before) [13] is yet to be explored.

In this study, we investigated the (1) diagnostic yield of SEP for PVC detection through a direct comparison with the 24-hour Holter monitoring and (2) variations in the PVC burden recorded during the 3-day extended monitoring using SEP and the associated clinical factors. Ultimately, we aimed to explore the potential clinical utility of SEP as a more convenient diagnostic tool of PVC, with the goal of identifying more patients who have clinically significant PVC burden.

## Methods

### Ethics Approval

The institutional review board at Seoul National University Hospital authorized this study (H-2103-010-1201). All study participants provided written informed consent. This study is reported according to the STROBE (Strengthening the Reporting of Observational Studies in Epidemiology) guidelines (Multimedia Appendix 1).

### Study Design and Patient Selection

This is a single-center and prospective cohort study of patients with PVC, which was performed between May 2021 and June 2022. Patients (≥19 years old) with documented PVC on a 12-lead ECG and scheduled for evaluation of the PVC burden (percentage of number of PVCs over the total QRS complexes) by 24-hour Holter monitoring were included. Since SEP utilizes a smartphone app to transmit ECG data, those unable to operate and manipulate the app were excluded. Three electrophysiologists (Choi EK, Lee SR, and SO) screened and recruited eligible patients at the outpatient clinic. Baseline clinical features, including demographic data, symptoms regarding PVC, comorbidities, lifestyle behaviors, medication history, and echocardiographic parameters were investigated.

### Validation of PVC Diagnosis by SEP Through Comparison With the Holter Monitor

The 24-hour Holter monitor (SEER Light, GE HealthCare) and SEP (mobiCARE MC-100, Seers Technology) record the electrical activity of the heart through 3 channels (leads I, V1, and V6) and a single channel (lead II), respectively. The process of validating the ECG algorithm for PVC detection by SEP and its performance are detailed in Multimedia Appendix 2. Patients underwent simultaneous ECG monitoring with the Holter monitor and SEP on the first day of monitoring. After 24 hours of monitoring, the Holter device was detached, and ECG monitoring was continued for the following 48 hours with SEP; the assessment of PVC burden using the Holter monitor is a part of the guideline-adherent practice [14], with patients covering the associated costs through local health insurance coverage. The detailed specifications of the SEP and the attachment methods for both devices are described in our prior report [15]. Information on total wear time, proportion of signal noise, total QRS complexes, and PVC burden was collected. An example strip of PVC detection using SEP is shown in Multimedia Appendix 3. For the evaluation of the diagnostic yield of PVC by SEP, the burdens of PVC assessed by the Holter monitor and SEP on the first day were compared with each other.

### Variability of PVC Burden and Clinical Factors Associated With Greater Variability

The fluctuation of PVC burden was assessed by the changes in the daily and 6-hour PVC burden monitored over a 3-day period using SEP. The purpose of evaluating daily and 6-hour PVC burden variations was to verify the fluctuations in the values. This analysis enables us to address the challenge of identifying the accurate overall PVC burden, and consequently, determining the appropriate treatment strategy. We defined daily PVC burden variation as the difference between the maximum and the minimum daily PVC burden measured by the SEP. The 6-hour PVC burden was calculated as the burden of PVC from midnight to 6 AM, 6 AM to noon, noon to 6 PM, and 6 PM to midnight. Similarly, the 6-hour PVC burden variation was defined as the difference between the maximum and minimum 6-hour PVC burden. We estimated the proportion of patients who were additionally identified to reach a clinically significant daily PVC burden (10%, 15%, or 20%) [12] during the 3-day monitoring. Further, clinical factors associated with a greater variation in PVC burden and those additionally influencing the PVC burden to cross the clinically meaningful threshold during the extended monitoring were investigated.

### Self-Reported Questionnaire About the Experience of the 3-Day Extended ECG Monitoring

The participants’ clinical experience of the 3-day extended ECG monitoring for PVC detection by SEP was evaluated using a self-reported questionnaire. The survey was conducted on a voluntary basis for all the study participants without offering any form of compensation on the day the SEP device was returned. Questions were based on the overall convenience of the Holter monitor and SEP, any instances of unexpected reattachment, and accessibility and user friendliness of mobile apps for SEP. Participants responded to the questions by using a scale ranging from 1 to 5 (1=no, 2=minimally, 3=sometimes, 4=likely, or 5=very likely).

### Statistical Analysis

Continuous variables were reported as mean (SD) or median (IQR) and compared by Student 2-sided *t* test or Mann-Whitney test. Categorical variables were presented as n (%), and Pearson chi-square test or Fisher exact test were applied as required. The diagnostic yield of PVC by SEP was compared to that by the Holter monitor with Bland-Altman plots with limits of agreement and scatter plots. The degree of agreement for the burden of PVC monitored by each device was analyzed. The associations between individuals’ baseline characteristics and the additional identification of signiﬁcant PVC thresholds (10%, 15%, or 20%) during the extended monitoring were evaluated by multivariable logistic regression analysis. All analyses were performed using Stata (version 17, StataCorp LLC).

## Results

### Baseline Characteristics of the Patients

Of the data of 156 enrolled patients with documented PVC on a 12-lead ECG, the data of 13 patients could not be retrieved due to errors in their smartphones, unfamiliarity with app use, or missing telemetry data transmission of SEP. Nine patients withdrew from the examination due to discomfort and skin irritability: 5 patients due to frequent alarms and the requirement of keeping their smartphones in close proximity, 2 due to skin irritability, and 2 due to inexperienced operation of the app. Finally, data from 134 patients with enough wear time of the patch (≥4200 minutes) and whose data were transmitted completely were included in this analysis.

The baseline characteristics of the included patients are described in Table 1. The mean age was 54.6 (SD 13.3) years, and 45 (33.6%) patients were males. The majority of the patients reported symptoms such as palpitation (96/134, 71.6%) or dizziness (15/134, 11.2%). Hypertension (46/134, 34.3%) and diabetes mellitus (21/134, 15.7%) were the common comorbidities. Congestive heart failure and cardiomyopathy were reported in 5.9% (8/134) and 2.9% (4/134) of the patients, respectively. Patients frequently took β-blockers (97/134, 72.4%) or calcium channel blockers (10/134, 7.5%) to relieve symptoms and PVC burden.

**Table 1:** Baseline characteristics of the patients with premature ventricular complex (N=134).

			Values
Age (years), mean (SD)			54.6 (13.3)
Sex (male), n (%)			45 (33.6)
BMI (kg/m^2^), mean (SD)			24.3 (3.4)
**Symptoms, n (%)**			
	Palpitation	96 (71.6)	
	Syncope	3 (2.2)	
	Dizziness	15 (11.2)	
**Comorbidities, n (%)**			
	Hypertension	46 (34.3)	
	Diabetes mellitus	21 (15.7)	
	Congestive heart failure	8 (5.9)	
	Cardiomyopathy	4 (2.9)	
	Coronary artery disease	0 (0)	
	Hypo/hyperthyroidism	5 (3.7)	
	Stroke/transient ischemic attack/thromboembolism	3 (2.2)	
**Lifestyle behaviors, n (%)**			
	Current smoking	8 (5.9)	
	Alcohol intake	16 (11.9)	
**Medications, n (%)**			
	β-blocker	97 (72.4)	
	Calcium channel blocker	10 (7.5)	
	Amiodarone	3 (2.2)	
	Class1c antiarrhythmic drug	3 (2.2)	
	Renin-angiotensin-aldosterone system blockade	20 (14.9)	
	Diuretics	5 (3.7)	
	Statin	29 (21.6)	
	Antiplatelet	15 (11.2)	
Ejection fraction (%), mean (SD)			58.8 (6)

### Diagnostic Performance of PVC Detection and Burden by SEP Compared to That by the Holter Monitor

An example of PVC detection by the adhesive SEP is presented in Multimedia Appendix 3. Detailed parameters of monitoring and the overall burden of PVC were compared between the 2 monitoring methods (Holter monitor and SEP) and are summarized in Table 2. The total mean wear time was 1395.1 (SD 33.5) minutes for the Holter monitor and 4318.0 (SD 12.4) minutes for SEP. The transmitted data were retrieved without significant noise for both devices (0.1% for the Holter monitor vs 0.9% for SEP). The median PVC burden of all the enrolled patients was 2.4% (IQR 0.2%-10.9%), as measured by the Holter monitor, and 3.3% (IQR 0.3%-11.7%), as measured by SEP during the 3-day extended monitoring, without significant difference (*P*=.58). During the first 24-hour monitoring by SEP, the burden of PVC among all patients was 7.5% (SD 10%; median 2.8%, IQR 0.3%-11%) without a significant difference from the PVC burden measured by the Holter monitor (*P*=.24). For those with PVC burden ≥5%, the overall PVC burden was detected as 13.3% (IQR 8%-24%) by the Holter monitor and 13.2% (IQR 9.7%-20.5%) by SEP during the 3-day extended monitoring (*P*=.10). During the first 24-hour monitoring by SEP, the burden of PVC among patients with a burden ≥5% was 16.5% (SD 9.9%; median 13.7%, IQR 8.1%-24.4%); the *P* value compared to the PVC burden detected by the Holter monitor was .82 (Table 2).

**Figure 1:**
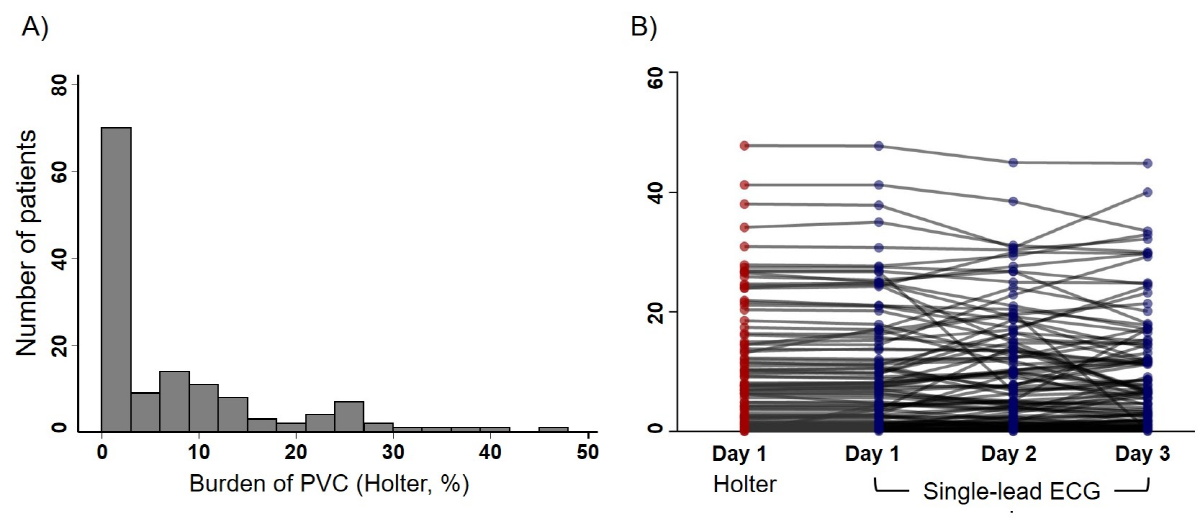
(A) Distribution of the patients with premature ventricular complex. (B) Comparison of the burden of premature ventricular complex evaluated by the Holter monitor and the adhesive single-lead electrocardiogram patch. ECG: electrocardiogram; PVC: premature ventricular complex.

**Figure 2:**
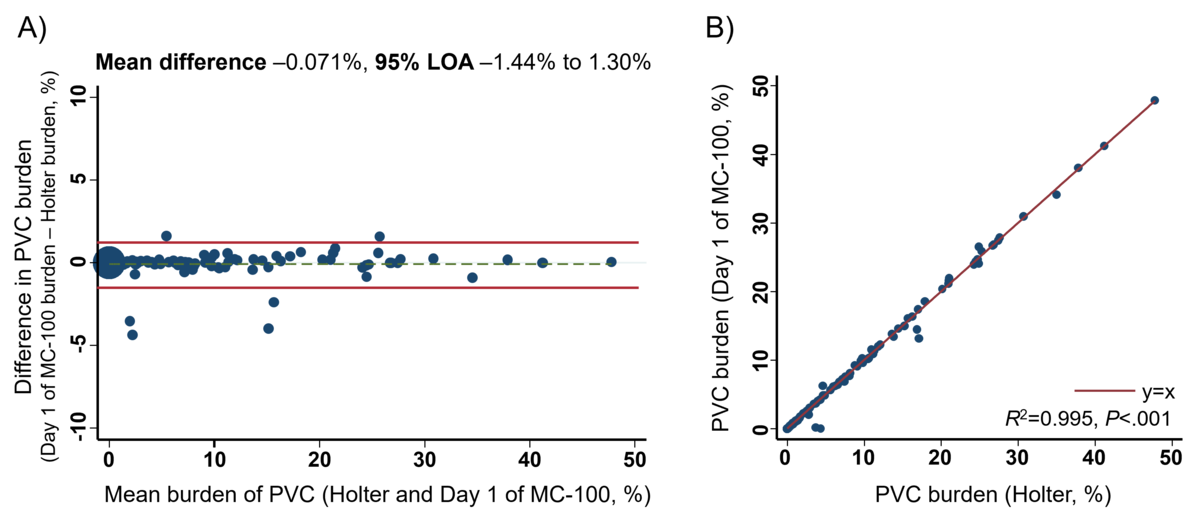
Agreement for the premature ventricular complex burden between Holter monitoring and adhesive single-lead electrocardiogram patch monitoring (mobiCARE MC-100) on day 1 analyzed by (A) Bland-Altman plot with limits of agreement and (B) scatter plot. LOA: limits of agreement; PVC: premature ventricular complex.

The overall distribution of the patients with PVC burden is presented in Figure 1A. Most of the patients (97/134, 72.4%) had a PVC burden <10%. PVC burden was reported as 10%-19.9% and ≥20% in 18 (13.4%) and 19 (14.2%) patients, respectively. An individual comparison of patients’ PVC detection by the Holter monitor and SEP is shown in Figure 1B. The PVC burden evaluated by the Holter monitor was repeated with almost the same value as the PVC burden estimated by SEP on the first day, whereas PVC burden variation was observed across days on SEP (Figure 1B). The validation of the diagnostic yield of SEP was performed by Bland-Altman analysis, and high agreement of PVC detection between the Holter monitor and SEP was confirmed with a mean difference of –0.07% and 95% limit of agreement of –1.44% to 1.30% (Figure 2A). PVC burdens detected by the Holter monitor and SEP on the first day of monitoring were highly correlated with each other (Figure 2B, *R*^2^=0.995), indicating the accurate detection of PVC by SEP.

**Table 2:** Evaluation of premature ventricular complex by the Holter monitor and the adhesive single-lead electrocardiogram patch (N=134).

		Holter monitor	Single-lead ECG^a^ patch	*P* value
Wear time (min), mean (SD)		1395.1 (33.5)	4318 (12.4)	N/A^b^
Noise (%), median (IQR)		0.1 (0.1-0.1)	0.9 (0.4-2.3)	<.001
Average heart rate (bpm), mean (SD)		71.1 (8.3)	72 (7.8)^c^	.01
Minimum heart rate (bpm), mean (SD)		52.1 (39.5)	49.1 (23.6)^c^	.44
Maximum heart rate (bpm), mean (SD)		122.4 (18.1)	131.8 (20.8)^c^	<.001
**Premature ventricular complex information**				
	Patients with PVC^d^ burden ≥5%, n (%)	56 (41.8)	57 (42.5)	<.001
	Patients with PVC burden ≥10%, n (%)	37 (27.6)	38 (28.4)	<.001
	Patients with PVC burden ≥15%, n (%)	23 (17.2)	22 (16.4)	<.001
	Burden of PVC among all patients (%), mean (SD); median (IQR)	7.4 (10); 2.4 (0.2-10.9)	7.3 (9.4)^e^; 3.3 (0.3-11.7)^e^	.58
	Burden of PVC among patients with burden ≥5% (%), mean (SD); median (IQR)	16.4 (9.9); 13.3 (8-24)	15.8 (9.3)^f^; 13.2 (9.7-20.5)^f^	.10

^a^ECG: electrocardiogram.

^b^N/A: not applicable.

^c^During the first 24-hour monitoring with the single-lead electrocardiogram patch, the average, minimum, and maximum heart rates (bpm) were 72.2 (SD 8.3), 48.8 (SD 6.2), and 123.2 (SD 17.7), respectively. The *P* values with the comparison of each value with the Holter monitor were <.001, .04, and .002, respectively.

^d^PVC: premature ventricular complex.

^e^During the first 24-hour monitoring with the single-lead electrocardiogram patch, the burden of premature ventricular complex among all patients was 7.5% (SD 10%; median 2.8%, IQR 0.3%-11%). The *P* value with the comparison of premature ventricular complex burden detected on the Holter monitor was .24.

^f^During the first 24-hour monitoring with the single-lead ECG patch, the burden of premature ventricular complex among patients with burden ≥5% was 16.5% (SD 9.9%; median 13.7%, IQR 8.1%-24.4%). The *P* value with the comparison of premature ventricular complex burden detected on the Holter monitor was .82.

### Variability of PVC Burden and Clinical Factors Associated With Greater Variability

**Figure 3:**
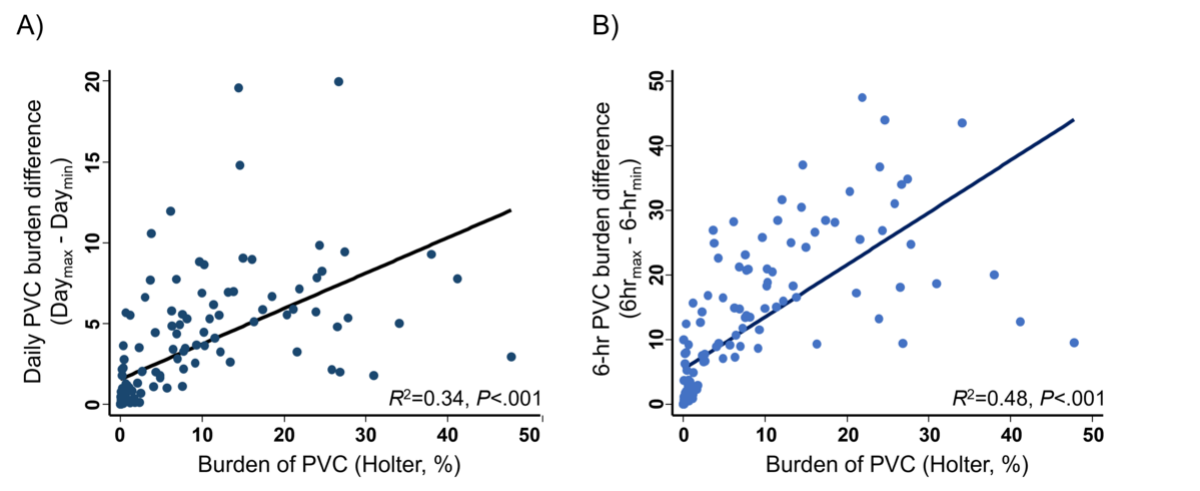
Correlation of the premature ventricular complex burden with (A) daily burden variation and (B) 6-hour burden variation. PVC: premature ventricular complex.

PVC burden varied widely from day to day and hour to hour, as shown in Figure 1B. Greater variations in PVC burden were observed when evaluated every 6 hours. In the same patient, the maximum daily PVC burden was 1.68-fold (IQR 1.31-3.02) higher than the minimum daily burden and the maximum 6-hour burden was 12.08-fold (IQR 4.00-57.50) higher than the minimum 6-hour burden; for those with PVC burden ≥1%, the maximum daily and 6-hour PVC burden were 1.52-fold (IQR 1.28-2.07) and 12.17-fold (IQR 5.06-49.46) higher than each minimum burden, respectively. Moreover, the variation was dependent on the individuals’ burden value; patients with higher PVC burden exhibited greater daily or hourly burden variation. Daily and 6-hour burden variations according to the value of PVC burden are presented in Figure 3, showing a moderate to high degree of correlation (*R*^2^=0.337 for daily and 0.483 for 6-hour burden variation). An example of an ECG strip of an individual presenting a high variation in daily and 6-hour PVC burden monitored using SEP is shown in Multimedia Appendix 4.

**Figure 4:**
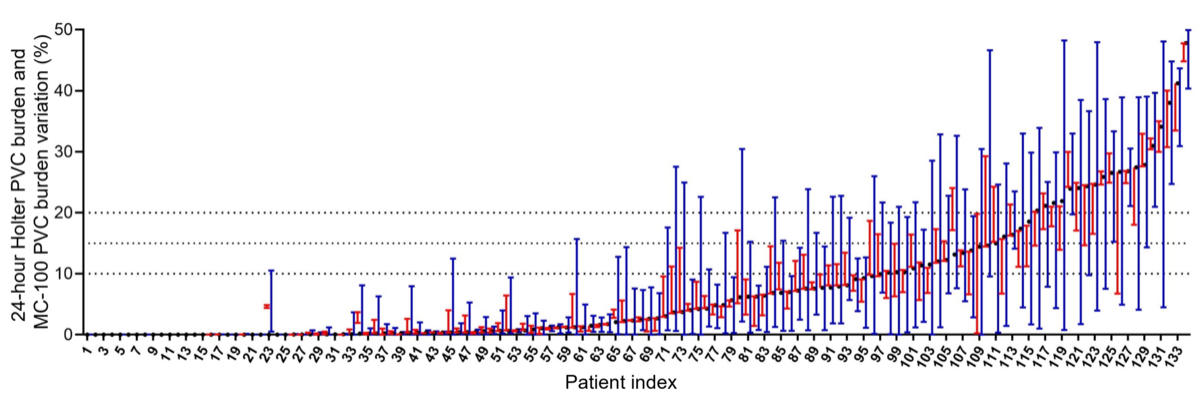
Patients additionally identified to reach the daily premature ventricular complex burden of 10%, 15%, and 20% during the 3-day monitoring with the adhesive single-lead electrocardiogram patch (mobiCARE MC-100). PVC: premature ventricular complex.

The number of patients with PVC burden lower than the clinically significant thresholds was 42 for 10%, 56 for 15%, and 60 for 20%. However, the 3-day extended monitoring additionally identified that 29% (12/42), 18% (10/56), and 7% (4/60) of the patients reached 10%, 15%, and 20% thresholds of the PVC burden, respectively (Figure 4, Table 3).

The clinical features of those crossing the PVC burden cutoff of 10%, 15%, or 20% during the 3-day extended monitoring were compared with those of the other patients with PVC burden lower than the clinically significant thresholds (Multimedia Appendix 5). Patients who were additionally identified to reach clinically significant thresholds during the 3-day extended monitoring were younger (*P*=.03), had congestive heart failure (*P*=.01) or cardiomyopathy (*P*=.01), and were using amiodarone (*P*=.04). When multivariable logistic regression analysis was performed, young age was independently associated with possible additional detection of PVC burden with clinical significance (Table 4; β=.95, 95% CI .92-.99; *P*=.02 for age).

**Table 3:** Patients additionally identified to reach the daily premature ventricular complex burden of 10%, 15%, and 20% during the 3-day monitoring with the adhesive single-lead electrocardiogram patch.

PVC^a^ threshold	Patients with PVC burden lower than the threshold (n)	Patients identified to reach the threshold during the 3-day monitoring with SEP^b^, n (%)	
		Daily burden	6-hour burden
10%	42	12 (29)	25 (60)
15%	56	10 (18)	31 (55)
20%	60	4 (7)	24 (40)

^a^PVC: premature ventricular complex.

^b^SEP: single-lead electrocardiogram patch.

**Table 4:** Clinical factors associated with additional detection of premature ventricular complex burden of thresholds 10%, 15%, and 20%.

Variable	Univariable analysis			Multivariable analysis	
	Odds ratio (95% CI)	*P* value	β (95% CI)		*P* value
Age	0.961 (0.928-0.996)	.03	.95 (.92-.99)		.02
Congestive heart failure	6.875 (1.562-30.256)	.01	N/A^a^		N/A
Cardiomyopathy	19.94 (1.960-202.893)	.01	N/A		N/A
Amiodarone	12.556 (1.082-145.709)	.04	N/A		N/A
Burden of premature ventricular complex	1.024 (0.982-1.069)	.27	N/A		N/A

^a^N/A: not applicable.

### Patients’ Experience of 3-Day Extended ECG Monitoring

A survey on patients’ experience with Holter monitoring and SEP was completed by 124 patients (response rate, 124/134, 92.5%), and the results are detailed in Multimedia Appendix 6. Overall, patients experienced lesser discomfort and lesser skin irritability with SEP compared to that with the Holter monitor (*P*<.001 and *P*=.02, respectively), but they wished to stop monitoring with SEP due to the longer duration of the examination. Indeed, since the patients wished to stop monitoring with SEP due to the longer duration of the examination, they detached the SEP and reattached the SEP later, thereby resulting in more instances of reattachment of the SEP (14/112, 12.5% for the Holter monitor and 66/118, 55.9% for SEP; *P*=.02). Thus, patients were more favorable to using SEP than the Holter monitor; 75.7% (84/111) of the patients responded to choosing a patch monitor for their next examination.

## Discussion

### Principal Results

Our principal findings are as follows: (1) SEP can diagnose PVC with comparable accuracy to the 24-hour Holter monitor, (2) the wide variation in the PVC burden recorded by SEP was proportional to the overall PVC frequency, (3) 3-day extended monitoring could identify those who reach clinically significant cutoffs of PVC burden, and (4) young age is associated with additional detection of clinically significant burdens of PVC during extended monitoring.

### Comparison to Prior Work

Ambulatory ECG monitoring is the principal diagnostic tool for evaluating PVC burden, and recently developed wearable ECG monitoring devices enable extended monitoring for more than 24 hours [1,16,17]. In particular, SEP is considered to be a sufficient and practical method to evaluate the PVC burden accurately and comprehend the wide variations daily [11,18]. Previous studies [19,20] have demonstrated variations in the PVC burden to up to 23% change across days, while a recent study [10] has reported a 2.5-fold median difference between the maximum and minimum 24-hour PVC burden in the same patient over a 14-day monitoring period. Regarding the optimal duration of PVC monitoring, the median time to detect one’s maximum PVC burden was reported as 6 (IQR 2-11) days [12], and the ideal duration for accurate PVC burden assessment was suggested as 7 days [13]. Although the monitoring period in our study (3 days) was shorter than the observation period in previous reports [12,13], we still observed a considerable intraindividual median daily PVC burden difference of 1.7-fold. Given that the optimal period for monitoring PVCs has not been established and a long recording length could be burdensome for both patients and clinicians, we suggest a compromise in the monitoring length (3 days), thereby providing an acceptable level of daily PVC burden, which may improve patients’ compliance and adherence. Despite the general comfort of SEP, our survey results revealed that patients felt more likely to discontinue monitoring as the monitoring time became longer. Furthermore, an extended monitoring duration could potentially exacerbate patient discomfort and fatigue because of the requirement of carrying their mobile phones for data transmission. The discomfort could also be intensified by the alarm systems and skin irritability. In our study, 9 patients withdrew from the examination due to these practical challenges; thus, optimizing the monitoring duration could potentially decrease the dropout rate associated with these issues.

A wide variation in the daily PVC burden detected during the 3-day monitoring enabled us to additionally identify 6.7%-28.6% of the patients who reach clinically significant thresholds (10%, 15%, or 20%). Compared to a previous study [12] that showed that extended monitoring nearly doubled the identification of those reaching the 10% threshold during 14-day monitoring, the low proportion of identification in our study could be due to the shorter duration of monitoring (3 days). However, this finding is consistent with the fact that extended monitoring brings incremental gain in identifying patients reaching the significant cutoffs of daily PVC burden. We observed that SEP could diagnose PVC with almost the same accuracy as the Holter monitor, and 3-day monitoring could detect patients who might benefit from an advanced treatment modality such as radiofrequency catheter ablation.

### Implications for Practice

Several risk factors are known to be associated with the presence of PVC and higher PVC frequency. Increased age, taller height, higher blood pressure, and unhealthy lifestyle behaviors such as smoking and less physical activity are consistently shown to be associated with the presence or higher frequency of PVC [21-23]. However, no study has demonstrated the clinical factors related to the large variations in PVC burden, which might be necessary to stratify patients requiring extended monitoring for a more accurate assessment of PVC burden. We found that the PVC burden evaluated during the first 24-hour monitoring was correlated not only with the day-to-day but also with the hour-to-hour variation. The higher frequency of PVCs was linked to a wider variation in the overall burden. In addition, we found that younger age, presence of congestive heart failure or cardiomyopathy, and use of amiodarone are clinical factors that may increase the likelihood that PVC burden reaches clinically significant thresholds during the 3-day extended monitoring. Above all, multivariable analysis showed that younger age was consistently associated with the significant variations in the PVC burden (*P*=.02). Although no explainable mechanism for this association has been reported yet, the fact that older age and a lack of diurnal variation of PVC frequency are related to a higher risk of PIC [24,25] imply that there might be a link between young age and a greater variation in the PVC burden. Our analyses revealed patients who required extended monitoring for a more precise evaluation of PVC burden, thus improving the determination of the treatment strategy. The variable circadian distribution of PVC burden and its associated clinical factors can provide additional value, as this information can be used to guide the pharmacologic induction of PVCs during radiofrequency catheter ablation and predict the outcome in patients [25].

### Strengths and Limitations

Our study has several limitations. First, the diagnostic yield of PVC via SEP and the degree of variation monitored for 3 days should be examined in a larger population. Second, since most of the included patients had normal ventricular systolic function, the accurate diagnosis of PVC and changes in PVC frequency monitored by SEP should be further validated in patients with reduced ejection fraction or structural heart diseases. Third, more than two-thirds of the patients had low PVC burden (<10%); thus, the extrapolation of our findings in patients with higher PVC burden (10%, 15%, or 20%) could be limited. Nonetheless, the distribution of the PVC burden in our study may represent the characteristics of patients in real-world settings, suggesting that our findings can be readily applied in a clinical context. Lastly, the external replication of our findings by using other wearable ECG patches in a multicenter setting would be required for the confirmation of generalizability.

### Conclusions

In a single-center prospective registry of patients with PVC, we validated that SEP can accurately diagnose PVC with almost the same yield as the 24-hour Holter monitor. We found that during the 3-day extended monitoring with SEP, the significant fluctuations in daily and 6-hour PVC burden were proportional to the overall PVC frequency. Further, 3-day extended monitoring of PVC by using SEP enabled the identification of more patients exceeding the clinically significant burden threshold; thus, SEP monitoring could be a practical method with acceptable patient adherence to enhance the decision on the optimal treatment strategy (ie, catheter ablation) for PVC. Several clinical factors, especially younger age, were associated with a higher variation of daily PVC burden, implying the necessity of extended monitoring for accurate assessments.

## Appendix

Multimedia Appendix 1 STROBE (Strengthening the Reporting of Observational Studies in Epidemiology) guidelines.

Multimedia Appendix 2 Process of validating the electrocardiogram algorithm for premature ventricular complex detection by single-lead electrocardiogram patch and its performance.

Multimedia Appendix 3 An example strip of premature ventricular complex detection by the single-lead electrocardiogram patch.

Multimedia Appendix 4 An example of an electrocardiogram strip of an individual presenting a high variation in daily and 6-hour premature ventricular complex burden monitored using a single-lead electrocardiogram patch.

Multimedia Appendix 5 Comparison of the clinical features of patients crossing the premature ventricular complex burden cutoffs of 10%, 15%, or 20% during the 3-day extended monitoring.

Multimedia Appendix 6 Survey completed by 124 patients on their experience with the Holter monitor and single-lead electrocardiogram patch.

## Abbreviations

ECG: electrocardiogram
PIC: premature ventricular complex–induced cardiomyopathy
PVC: premature ventricular complex
SEP: single-lead electrocardiogram patch
STROBE: Strengthening the Reporting of Observational Studies in Epidemiology

## Data Availability

All data generated or analyzed during this study are included in this paper and Multimedia Appendices 1-6. The data of this study will be shared upon reasonable request to the corresponding author.

## References

[1] Marcus GM (2020). Evaluation and management of premature ventricular complexes. Circulation.

[2] El Kadri M, Yokokawa M, Labounty T, Mueller G, Crawford T, Good E, Jongnarangsin K, Chugh A, Ghanbari H, Latchamsetty R, Oral H, Pelosi F, Morady F, Bogun F (2015). Effect of ablation of frequent premature ventricular complexes on left ventricular function in patients with nonischemic cardiomyopathy. Heart Rhythm.

[3] Kennedy HL, Whitlock JA, Sprague MK, Kennedy LJ, Buckingham TA, Goldberg RJ (1985). Long-term follow-up of asymptomatic healthy subjects with frequent and complex ventricular ectopy. N Engl J Med.

[4] Bigger JT, Fleiss JL, Kleiger R, Miller JP, Rolnitzky LM (1984). The relationships among ventricular arrhythmias, left ventricular dysfunction, and mortality in the 2 years after myocardial infarction. Circulation.

[5] Baman TS, Lange DC, Ilg KJ, Gupta SK, Liu T, Alguire C, Armstrong W, Good E, Chugh A, Jongnarangsin K, Pelosi F, Crawford T, Ebinger M, Oral H, Morady F, Bogun F (2010). Relationship between burden of premature ventricular complexes and left ventricular function. Heart Rhythm.

[6] Bogun F, Crawford T, Reich S, Koelling TM, Armstrong W, Good E, Jongnarangsin K, Marine JE, Chugh A, Pelosi F, Oral H, Morady F (2007). Radiofrequency ablation of frequent, idiopathic premature ventricular complexes: comparison with a control group without intervention. Heart Rhythm.

[7] Ban J, Park H, Park J, Nagamoto Y, Choi J, Lim H, Park S, Kim Y (2013). Electrocardiographic and electrophysiological characteristics of premature ventricular complexes associated with left ventricular dysfunction in patients without structural heart disease. Europace.

[8] Hasdemir C, Ulucan C, Yavuzgil O, Yuksel A, Kartal Y, Simsek E, Musayev O, Kayikcioglu M, Payzin S, Kultursay H, Aydin M, Can LH (2011). Tachycardia-induced cardiomyopathy in patients with idiopathic ventricular arrhythmias: the incidence, clinical and electrophysiologic characteristics, and the predictors. J Cardiovasc Electrophysiol.

[9] Latchamsetty R, Bogun F (2019). Premature ventricular complex-induced cardiomyopathy. JACC Clin Electrophysiol.

[10] Mullis AH, Ayoub K, Shah J, Butt M, Suffredini J, Czarapata M, Delisle B, Ogunbayo GO, Darrat Y, Elayi CS (2019). Fluctuations in premature ventricular contraction burden can affect medical assessment and management. Heart Rhythm.

[11] Voskoboinik A, Hadjis A, Alhede C, Im SI, Park H, Moss J, Marcus GM, Hsia H, Lee B, Tseng Z, Lee R, Scheinman M, Vedantham V, Vittinghoff E, Park K, Gerstenfeld EP (2020). Predictors of adverse outcome in patients with frequent premature ventricular complexes: The ABC-VT risk score. Heart Rhythm.

[12] Loring Z, Hanna P, Pellegrini CN (2016). Longer ambulatory ECG monitoring increases identification of clinically significant ectopy. Pacing Clin Electrophysiol.

[13] Hsia BC, Greige N, Patel SK, Clark RM, Ferrick KJ, Fisher JD, Gross J, Di Biase L, Krumerman A (2020). Determining the optimal duration for premature ventricular contraction monitoring. Heart Rhythm.

[14] Zeppenfeld K, Tfelt-Hansen J, de Riva M, Winkel BG, Behr ER, Blom NA, Charron P, Corrado D, Dagres N, de Chillou C, Eckardt L, Friede T, Haugaa KH, Hocini M, Lambiase PD, Marijon E, Merino JL, Peichl P, Priori SG, Reichlin T, Schulz-Menger J, Sticherling C, Tzeis S, Verstrael A, Volterrani M, ESC Scientific Document Group (2022). 2022 ESC Guidelines for the management of patients with ventricular arrhythmias and the prevention of sudden cardiac death. Eur Heart J.

[15] Kwon S, Lee S, Choi E, Ahn H, Song H, Lee Y, Oh S, Lip GYH (2022). Comparison between the 24-hour Holter test and 72-hour single-lead electrocardiogram monitoring with an adhesive patch-type device for atrial fibrillation detection: prospective cohort study. J Med Internet Res.

[16] Al-Khatib SM, Stevenson WG, Ackerman MJ, Bryant WJ, Callans DJ, Curtis AB, Deal BJ, Dickfeld T, Field ME, Fonarow GC, Gillis AM, Granger CB, Hammill SC, Hlatky MA, Joglar JA, Kay GN, Matlock DD, Myerburg RJ, Page RL (2018). 2017 AHA/ACC/HRS guideline for management of patients with ventricular arrhythmias and the prevention of sudden cardiac death: A Report of the American College of Cardiology/American Heart Association Task Force on Clinical Practice Guidelines and the Heart Rhythm Society. Heart Rhythm.

[17] Turakhia MP, Hoang DD, Zimetbaum P, Miller JD, Froelicher VF, Kumar UN, Xu X, Yang F, Heidenreich PA (2013). Diagnostic utility of a novel leadless arrhythmia monitoring device. Am J Cardiol.

[18] Heckbert SR, Austin TR, Jensen PN, Floyd JS, Psaty BM, Soliman EZ, Kronmal RA (2018). Yield and consistency of arrhythmia detection with patch electrocardiographic monitoring: the multi-ethnic study of atherosclerosis. J Electrocardiol.

[19] Winkle RA (1978). Antiarrhythmic drug effect mimicked by spontaneous variability of ventricular ectopy. Circulation.

[20] Morganroth J, Michelson EL, Horowitz LN, Josephson ME, Pearlman AS, Dunkman WB (1978). Limitations of routine long-term electrocardiographic monitoring to assess ventricular ectopic frequency. Circulation.

[21] von Rotz M, Aeschbacher S, Bossard M, Schoen T, Blum S, Schneider S, Estis J, Todd J, Risch M, Risch L, Conen D (2017). Risk factors for premature ventricular contractions in young and healthy adults. Heart.

[22] Kerola T, Dewland TA, Vittinghoff E, Heckbert SR, Stein PK, Marcus GM (2018). Modifiable predictors of ventricular ectopy in the community. J Am Heart Assoc.

[23] Simpson RJ, Cascio WE, Schreiner PJ, Crow RS, Rautaharju PM, Heiss G (2002). Prevalence of premature ventricular contractions in a population of African American and white men and women: the Atherosclerosis Risk in Communities (ARIC) study. Am Heart J.

[24] Bas HD, Baser K, Hoyt J, Yokokawa M, LaBounty T, Morady F, Bogun F (2016). Effect of circadian variability in frequency of premature ventricular complexes on left ventricular function. Heart Rhythm.

[25] Hamon D, Abehsira G, Gu K, Liu A, Blaye-Felice Sadron M, Billet S, Kambur T, Swid MA, Boyle NG, Dandamudi G, Maury P, Chen M, Miller JM, Lellouche N, Shivkumar K, Bradfield JS (2018). Circadian variability patterns predict and guide premature ventricular contraction ablation procedural inducibility and outcomes. Heart Rhythm.

