# Correction: Three-Day Monitoring of Adhesive Single-Lead Electrocardiogram Patch for Premature Ventricular Complex: Prospective Study for Diagnosis Validation and Evaluation of Burden Fluctuation

**DOI:** 10.2196/59984

**Published:** 2024-05-09

**Authors:** Hyo-Jeong Ahn, Eue-Keun Choi, So-Ryoung Lee, Soonil Kwon, Hee-Seok Song, Young-Shin Lee, Seil Oh

**Affiliations:** 1 Department of Internal Medicine Seoul National University Hospital Seoul Republic of Korea; 2 Department of Internal Medicine Seoul National University College of Medicine Seoul Republic of Korea; 3 Seers Technology Co, Ltd Seongnam-si Gyeonggi-do Republic of Korea

In “Three-Day Monitoring of Adhesive Single-Lead Electrocardiogram Patch for Premature Ventricular Complex: Prospective Study for Diagnosis Validation and Evaluation of Burden Fluctuation” (J Med Internet Res 2024;26:e46098) the authors noted one error.

In the “Ethics Approval” section, the institutional review board (IRB) number was originally published as:

E-2001-111-1096.

This has been changed to:

H-2103-010-1201.

The correction will appear in the online version of the paper on the JMIR Publications website on May 9, 2024, together with the publication of this correction notice. Because this was made after submission to PubMed, PubMed Central, and other full-text repositories, the corrected article has also been resubmitted to those repositories.

