# Supplementary material for: Three-Day Monitoring of Adhesive Single-Lead Electrocardiogram Patch for Premature Ventricular Complex: Prospective Study for Diagnosis Validation and Evaluation of Burden Fluctuation
Source: J Med Internet Res. 2024 Mar 21;26:e46098. doi: 10.2196/46098 (PMC10995782; doi:10.2196/46098)
Supplement: Multimedia Appendix 2 [file jmir_v26i1e46098_app2.docx]

**Multimedia Appendix 2.** Validation of ECG algorithm for PVC detection in SEP and its performance.

*Algorithm of adhesive single-lead ECG patch (SEP; mobiCARE Cardio, MC-100, Seers technology) to analyze electrocardiogram (ECG)*

It is a Python-based algorithm to detect QRS complexes, heart rate, arrhythmias, and other diagnostic parameters. The process is specifically performed using a single channel. The overall flow of the ECG analysis algorithm is described in **Figure 1**.

First, a deep learning semantic segmentation algorithm converts raw and unstructured ECG data into structured data dividing ECG components into baseline, P-wave, QRS complex, T-wave, noise, and fibrillation probabilities on a per-sample basis. The training dataset was created using public datasets such as MIT-BIH, AHA, CU, NST, and internal clinical research data.^1,2^ We utilized DDR-Net for high accuracy and fast performance (**Figure 2**).^3^

Second, ECG feature extraction is performed using a rule-based algorithm based on standardized data obtained through segmentation. Peak detection is the process of finding the point with the largest absolute value in the central 1/2 area except for the region of both 1/4 ends in the region segmented by QRS. Noise detection is the process of connecting the area segmented by noise with a short interval and removing the noise section that is too short (**Figure 3**).

Third, heartbeat detection/classification is the process of distinguishing between the normal conducted QRS, premature ventricular contractions (PVCs), and premature atrial contractions (PACs) based on extracted features such as segmentation, RR interval, amplitude, and length obtained from the previous feature extraction.

Figure 1. Overview of ECG analysis
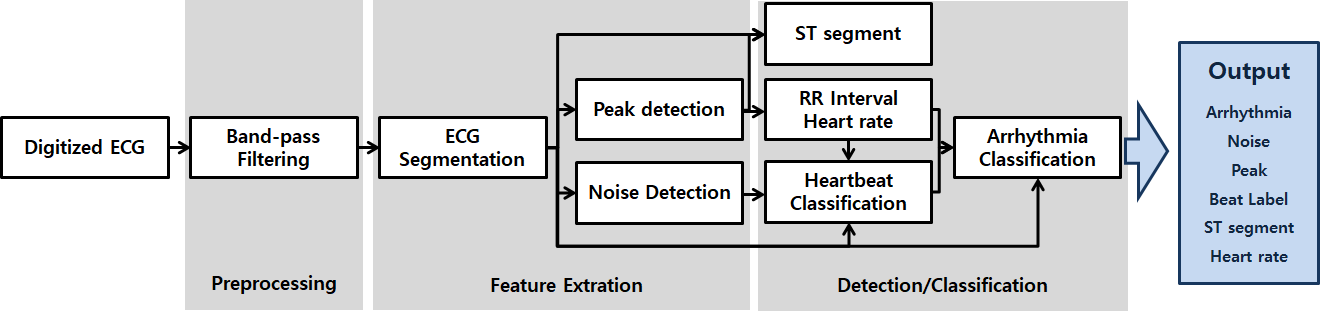


Figure 2. Standardized ECG data obtained through segmentation
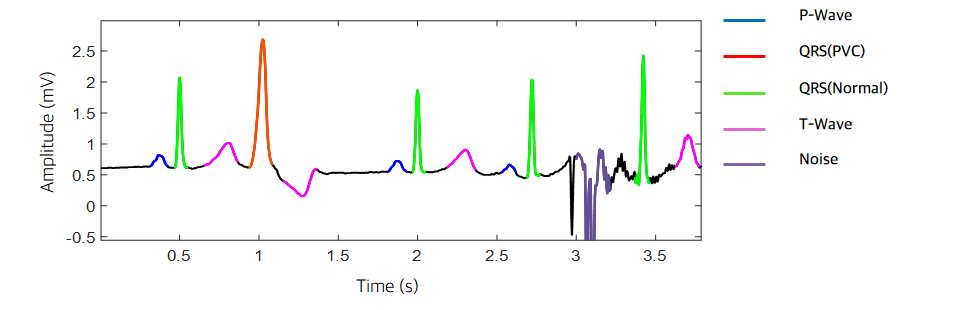


Figure 3. ECG feature extraction using a rule-based algorithm


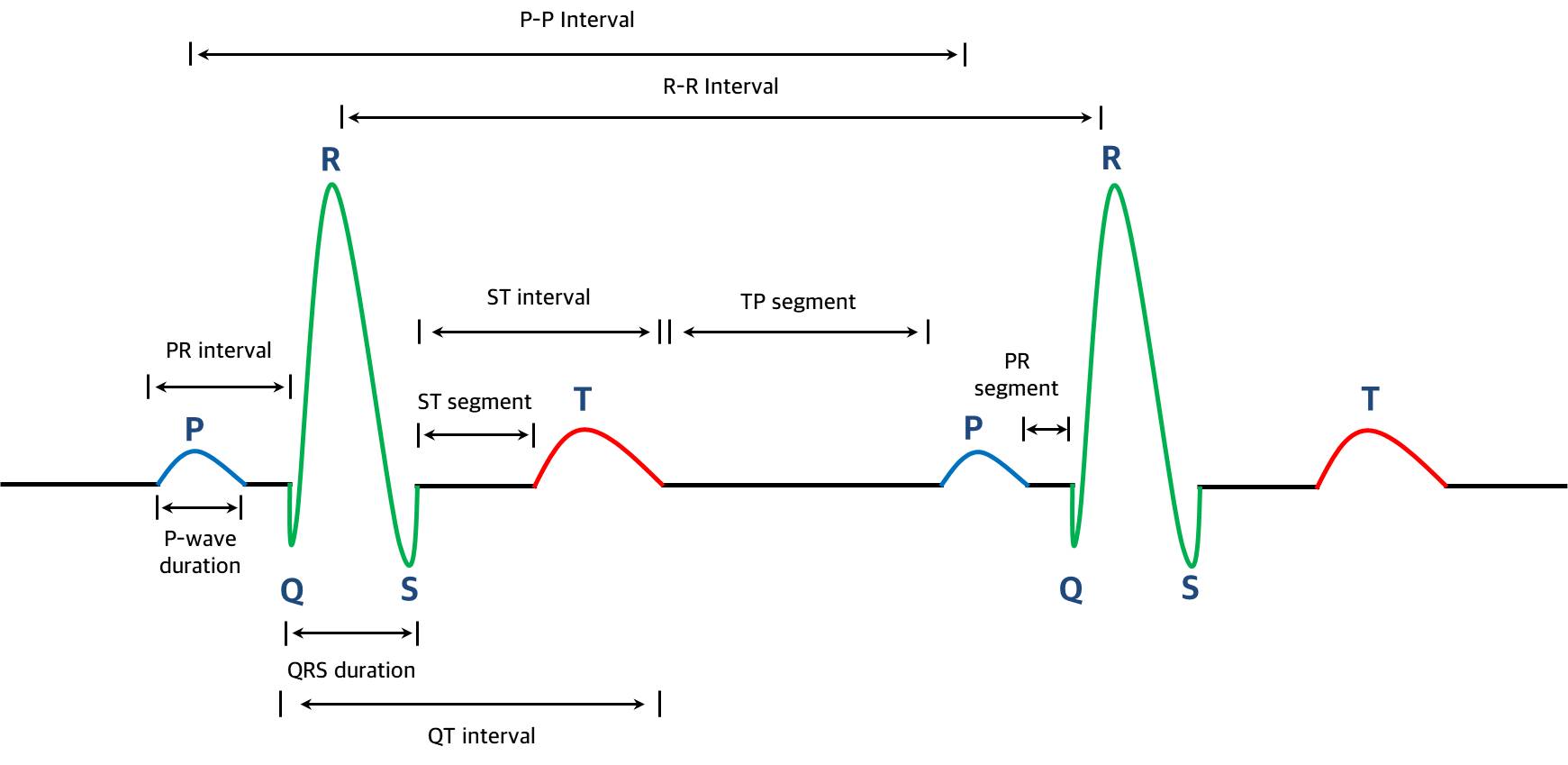


*Validation test of SEP (mobiCARE Cardio) for the detection of PVCs*

The test method of SEP performance detecting PVCs is summarized in **Figure 4.** The following standards, ECG test dataset, and evaluation equipment were utilized. The overall performance of the SEP in detecting QRS complex and PVCs is summarized in **Table 1**. The sensitivity of QRS complex detection is 99.2-99.7% with over 99.9% of positive predictive value (PPV). The sensitivity of PVC detection is 90.7-94.6%, with 90.5-91.6% PPV.

- Standards: IEC60601-2-47:2012
- ECG test dataset: MIT-BIH, AHA
- Evaluation equipment: WhaleTeq ECG Test System [Multi-Channel Test Unit (MECG), RDCA (Rhythm Database Compliance Analyzer) Database Comparison Software
- Evaluation parameters: sensitivity, positive predictive value, false positive rate.

Figure 4. Validation of SEP for the detection of PVCs
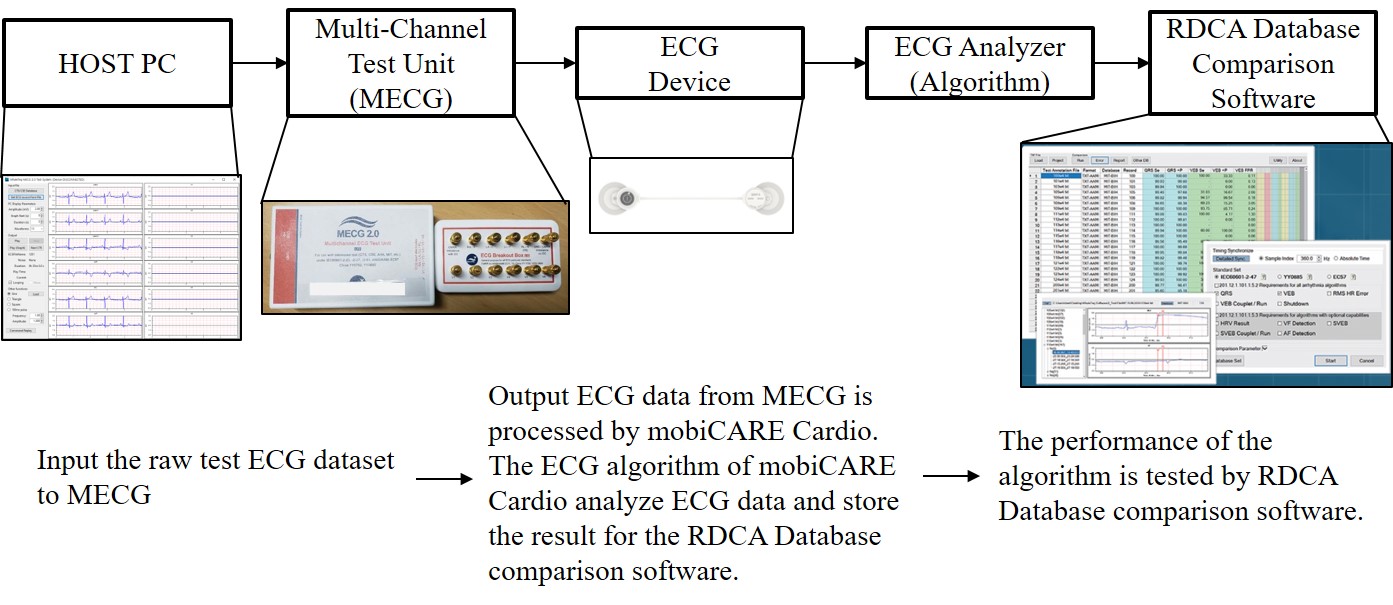


Table 1. The overall performance of the SEP (mobiCARE Cardio) to detect QRS complex and PVCs

|  | | ECG test dataset | |
| --- | --- | --- | --- |
|  |  | MIT-BIH | AHA database |
| QRS | Sensitivity | 99.68% | 99.18% |
|  | Positive predictive value | 99.99% | 99.98% |
| PVC | Sensitivity | 94.58% | 90.66% |
|  | Positive predictive value | 90.45% | 91.55% |
|  | False positive rate | 0.04% | 0.15% |

*Comparison of the classification of heartbeats detected on Holter and single-lead ECG patch (mobiCARE Cardio) among the study population (n=134)*

The high sensitivity and PPV of SEP used in our study to analyze ECG and interpret PVCs were also reproduced when compared to the Holter signals in the real-world clinic experience. We compared the number of total QRS complexes, ventricular beats, and supraventricular beats of the participants detected on Holter and the first day of SEP (the day when both devices were attached simultaneously in the same individual). As described in **Figure 5**, there was a high degree of agreement in all three categories of the heart beats; R^2^ was 0.828, 0.992, and 0.979 for the total QRS complexes, ventricular beats, and supraventricular beats, respectively.

The PVC burden detected on the Holter and that of the first day of SEP (simultaneous attachment) was also compared. We dichotomized PVC burden with cutoffs of 3%, 5%, and 10%. For each cutoff, the sensitivity, specificity, positive predictive value (PPV), negative predictive value (NPV), area under the receiver operating characteristic curve (AUC), and F1 score of SEP were compared to 24-hour Holter. As presented in **Table 2**, the PVC burden detected by SEP presented high sensitivity and specificity (97.10-100.00%) compared to that of 24-hour Holter with AUC 0.98-0.99. The high values represent a nearly identical diagnostic yield of PVC by SEP compared to that detected by 24-hour Holter.

Figure 5. The number of A) total QRS complexes, B) ventricular beats, and C) supraventricular beats detected on the Holter and the first day of single-lead ECG patch.


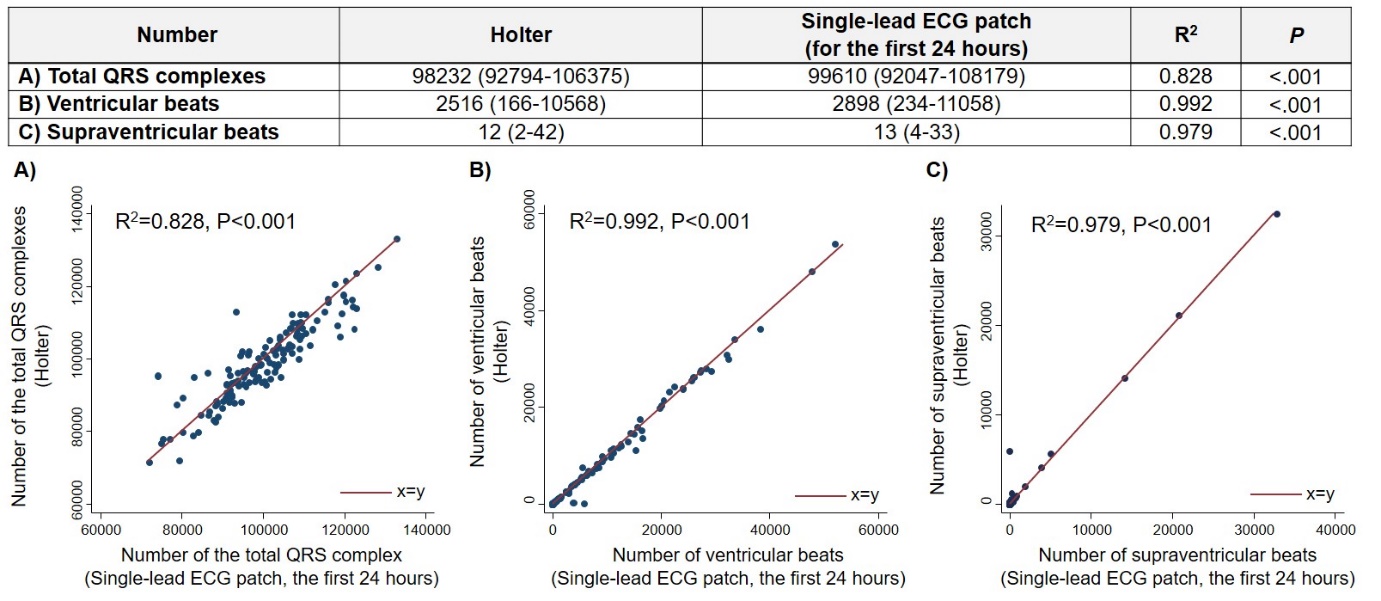


Table 2. The sensitivity, specificity, positive predictive value, negative predictive value, the receiver operating characteristic curve, and F1 score of the PVC burden detection by SEP compared to 24-hour Holter for various cutoff.

| The cutoff of PVC burden detected on the first day of SEP. | Sens. | Spec. | PPV | NPV | AUC | F1 score |
| --- | --- | --- | --- | --- | --- | --- |
| ≥3% | 98.44% | 97.10% | 96.92% | 98.53% | 97.77% | 0.98 |
| ≥5% | 98.21% | 100.00% | 100.00% | 98.72% | 99.11% | 0.99 |
| ≥10% | 97.30% | 100.00% | 100.00% | 98.97% | 98.65% | 0.99 |

Abbreviations: Sens., sensitivity; Spec., specificity; PPV, positive predictive value; NPV, negative predictive value; AUC, area under the receiver operating characteristic curve; SEP, single-lead ECG patch.

**References**

1. Moody GB, Mark RG. The impact of the MIT-BIH arrhythmia database. IEEE Eng Med Biol Mag. 2001 May-Jun;20(3):45-50. doi: 10.1109/51.932724. PMID: 11446209.
2. Goldberger AL, Amaral LA, Glass L, Hausdorff JM, Ivanov PC, Mark RG, Mietus JE, Moody GB, Peng CK, Stanley HE. PhysioBank, PhysioToolkit, and PhysioNet: components of a new research resource for complex physiologic signals. Circulation. 2000 Jun 13;101(23):E215-20. doi: 10.1161/01.cir.101.23.e215. PMID: 10851218.
3. Yuandua Hong, Huihui Pan, Weichao Sun. Deep Dual-Resolution Networks for Real-Time and Accurate Semantic Segmentation of Traffic Scenes. IEEE TITS. 2022 Dec 21;24(3):3448-3460. doi: 10.1109/TITS.2022.3228042. INSPEC Accession number: 22686131
