# Supplementary material for: Three-Day Monitoring of Adhesive Single-Lead Electrocardiogram Patch for Premature Ventricular Complex: Prospective Study for Diagnosis Validation and Evaluation of Burden Fluctuation
Source: J Med Internet Res. 2024 Mar 21;26:e46098. doi: 10.2196/46098 (PMC10995782; doi:10.2196/46098)

**Multimedia Appendix 3.** An example strip of detecting premature ventricular complex by the adhesive single-lead electrocardiogram patch.


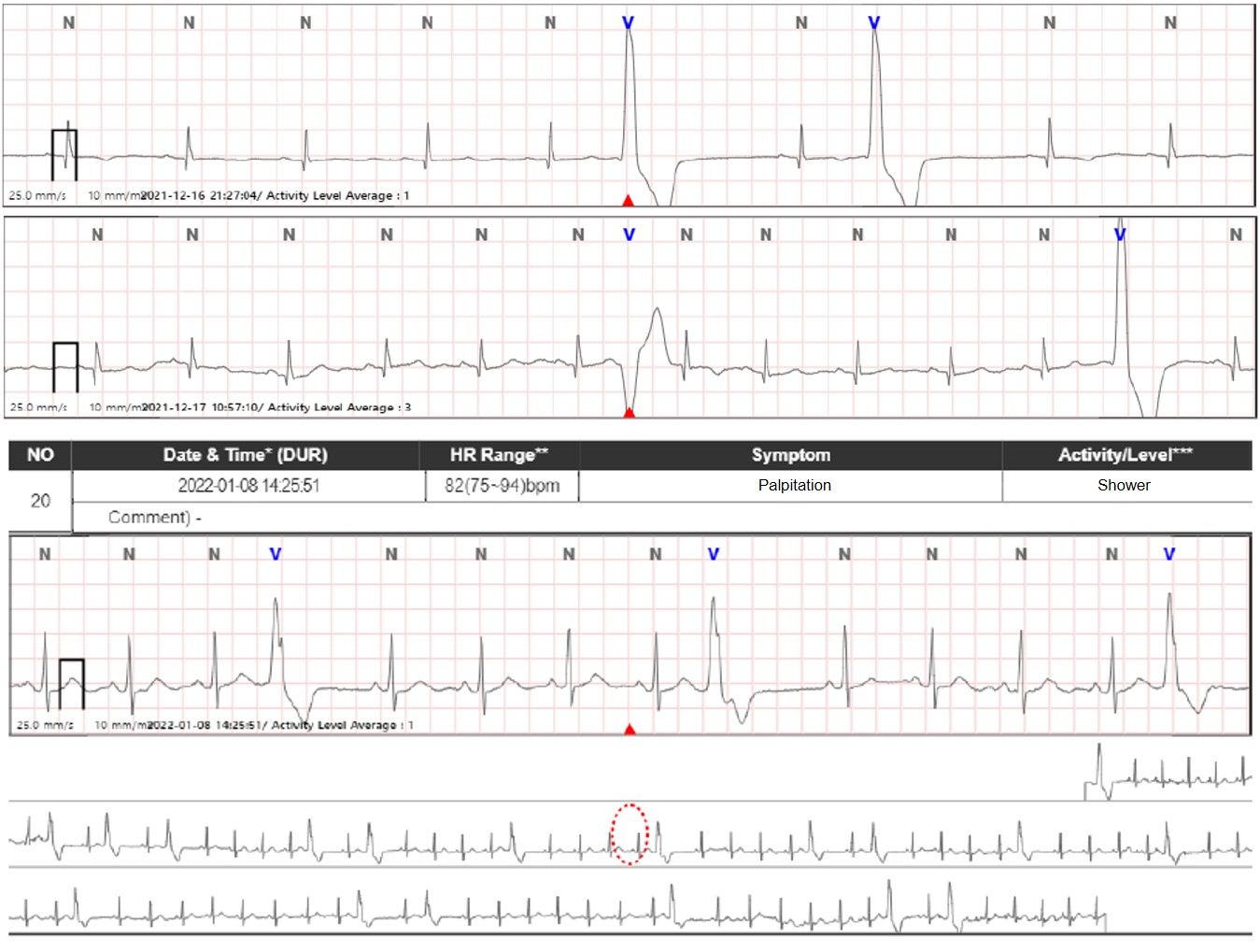

Supplement: Multimedia Appendix 3 [file jmir_v26i1e46098_app3.docx]
