# Supplementary material for: Three-Day Monitoring of Adhesive Single-Lead Electrocardiogram Patch for Premature Ventricular Complex: Prospective Study for Diagnosis Validation and Evaluation of Burden Fluctuation
Source: J Med Internet Res. 2024 Mar 21;26:e46098. doi: 10.2196/46098 (PMC10995782; doi:10.2196/46098)
Supplement: Multimedia Appendix 4 [file jmir_v26i1e46098_app4.docx]

**Multimedia Appendix 4.** Example of an individual presenting a high variation of daily and 6-hour premature ventricular complex burden monitored on the adhesive single-lead electrocardiogram patch.

Abbreviations: ECG, electrocardiogram; PVC, premature ventricular complex.

**
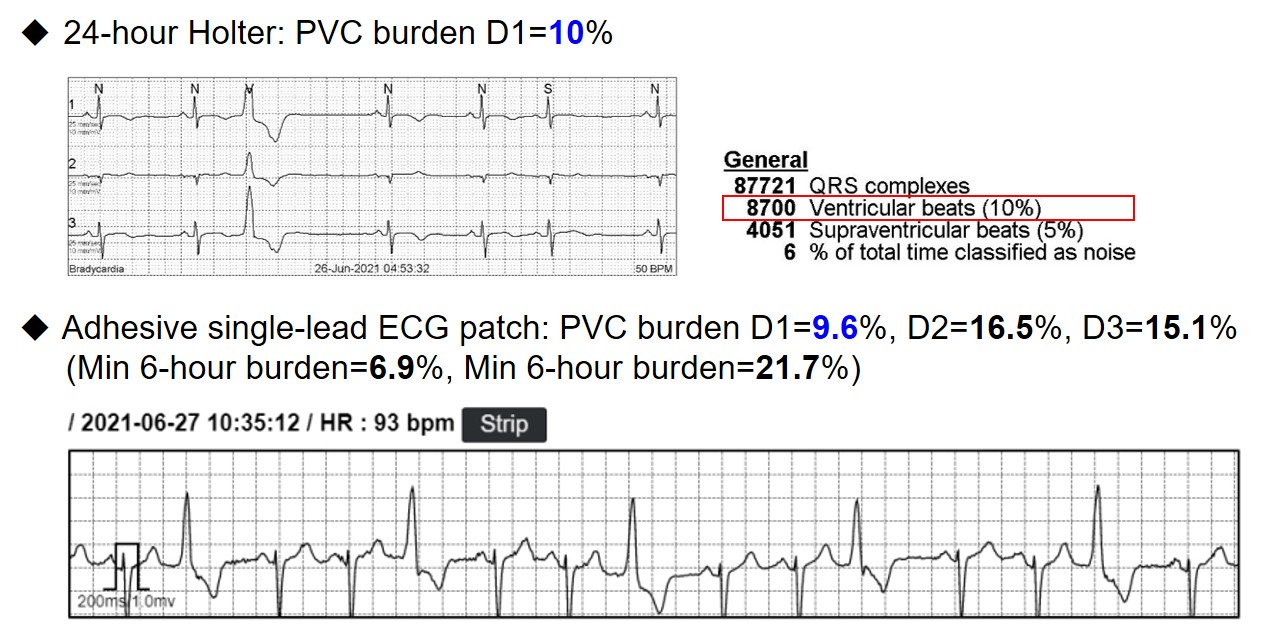
**
