# Supplementary material for: Three-Day Monitoring of Adhesive Single-Lead Electrocardiogram Patch for Premature Ventricular Complex: Prospective Study for Diagnosis Validation and Evaluation of Burden Fluctuation
Source: J Med Internet Res. 2024 Mar 21;26:e46098. doi: 10.2196/46098 (PMC10995782; doi:10.2196/46098)
Supplement: Multimedia Appendix 5 [file jmir_v26i1e46098_app5.docx]

**Multimedia Appendix 5.** Baseline characteristics of patients according to the additional detection of burden of premature ventricular complex over 10%, 15%, and 20%.

Abbreviations: TIA, transient ischemic attack; TE, thromboembolism; AAD, antiarrhythmic drug; RAAS, renin-angiotensin-aldosterone system.

|  | **Additional detection of PVC burden  threshold 10%, 15%, and 20%** | |  |
| --- | --- | --- | --- |
|  | **No (n=114)** | **Yes (n=20)** | ***P*** |
| **Age (years)** | 55.7±12.3 | 48.5±17.2 | 0.03 |
| **Sex (Male)** | 38 (33.3%) | 7 (35.0%) | 0.88 |
| **Body mass index (kg/m^2^)** | 24.2±3.3 | 24.9±4.0 | 0.36 |
| **Symptom** |  |  |  |
| Palpitation | 81 (71.1%) | 15 (75.0%) | 0.72 |
| Syncope | 3 (2.6%) | 0 (0.0%) | 0.46 |
| Dizziness | 14 (12.3%) | 1 (5.0%) | 0.34 |
| **Comorbidities** |  |  |  |
| Hypertension | 41 (36.0%) | 5 (25.0%) | 0.34 |
| Diabetes mellitus | 18 (15.8%) | 3 (15.0%) | 0.93 |
| Congestive heart failure | 1 (0.9%) | 4 (20.0%) | <.001 |
| Cardiomyopathy | 1 (0.9%) | 3 (15.0%) | <.001 |
| Coronary artery disease | 0 (0.0%) | 0 (0.0%) | N/A |
| Hypo/Hyperthyroidism | 4.0 (3.6%) | 1 (5.0%) | 0.56 |
| Stroke/TIA/TE | 3 (2.6%) | 0 (0.0%) | 0.46 |
| **Lifestyle behaviors** |  |  |  |
| Current Smoking | 8 (7.0%) | 0 (0.0%) | 0.22 |
| Alcohol intake | 12 (10.5%) | 4 (20.0%) | 0.23 |
| **Medications** |  |  |  |
| Beta-blocker | 81 (71.1%) | 16 (80.0%) | 0.41 |
| Calcium channel blocker | 7 (6.1%) | 3 (15.0%) | 0.16 |
| Amiodarone | 1 (0.9%) | 2 (10.0%) | 0.01 |
| Class1c AAD | 3 (2.6%) | 0 (0.0%) | 0.46 |
| RAASc blockade | 15 (13.2%) | 5 (25.0%) | 0.17 |
| Diuretics | 4 (3.5%) | 1 (5.0%) | 0.75 |
| Statin | 26 (22.8%) | 3 (15.0%) | 0.43 |
| Antiplatelet | 11 (9.6%) | 4 (20.0%) | 0.18 |
| **Ejection fraction (%)** | 59.4±5.3 | 55.4±8.5 | 0.01 |
