# Supplementary material for: Three-Day Monitoring of Adhesive Single-Lead Electrocardiogram Patch for Premature Ventricular Complex: Prospective Study for Diagnosis Validation and Evaluation of Burden Fluctuation
Source: J Med Internet Res. 2024 Mar 21;26:e46098. doi: 10.2196/46098 (PMC10995782; doi:10.2196/46098)
Supplement: Multimedia Appendix 6 [file jmir_v26i1e46098_app6.docx]

**Multimedia Appendix 6.** Self-reported questionnaire on the use of the adhesive single-lead electrocardiogram patch.

Abbreviations: ECG, electrocardiogram.

Response: 1. No; 2. Minimally; 3. Sometimes; 4. Likely; 5. Very likely.

|  | **Holter** | **Single-lead ECG** | ***P*** |
| --- | --- | --- | --- |
| Discomfort | 3.54±1.44 | 2.56±1.22 | <.001 |
|  | (4,5: 59.1%) | (4,5: 27.5%) |  |
| Skin irritability | 3.19±1.45 | 2.89±1.46 | 0.02 |
|  | (4,5: 51.6%) | (4,5: 41.3%) |  |
| Willingness to stop monitoring | 2.66±1.32 | 3.28±1.49 | <.001 |
|  | (4,5: 51.3%) | (4,5: 31.6%) |  |
| Reattachment experience | 14/112 (12.5%) | 66/118 (55.9%) | 0.02 |
| Easy to record symptoms (application for a single-lead ECG ) | N/A | 3.49 ± 1.36 | N/A |
|  |  | (21.0%-difficult) |  |
| Easy to use (overall evaluation) | N/A | 4.19 ± 1.02 | N/A |
|  |  | (5.8%-difficult) |  |
| Holter vs. Single-lead ECG | 27/111 (24.3%) | 84/111 (75.7%) | N/A |
